# Why not to treat the tooth canal to solve external root resorptions? Here are the principles!

**DOI:** 10.1590/2177-6709.21.6.020-025.oin

**Published:** 2016

**Authors:** Alberto Consolaro, Graziella Bittencourt

**Affiliations:** 1 Full professor at Faculdade de Odontologia de Bauru, Universidade de São Paulo (FOB-USP) and in the Post-Graduation program at Faculdade de Odontologia de Ribeirão Preto, Universidade de São Paulo (FORP-USP).; 2 Professor of Endodontics at ABO-ES and at ESFA-ES.

**Keywords:** Tooth resorption, Root resorption, External resorptions, Resorptions treatment

## Abstract

This paper aims at exposing the foundations or reasons why, in cases of external tooth resorption, including those of orthodontic origin, one should not perform a root canal to treat it. That should be done only to teeth with contamination or pulp necrosis, to remove the periapical inflammation induced by microbial products. When facing cases of external tooth resorption, one's conduct must always respect the following sequence of steps: first of all, identifying the cause accurately; then, planning the therapeutic approach and, finally, adopting the conducts in a very well-founded way. The situations in which endodontic treatment is recommended for tooth resorptions are those when there are: a) pulp necrosis with microbial contamination, b) aseptic pulp necrosis, c) developing calcific metamorphosis of the pulp and d) diagnosis of internal resorption. It is not possible, through the pulp, to control the resorption process that is taking place in the external part, after all, the causes are acting in the periodontal ligament. There is no evidence that justifies applying endodontic treatment, by means of root canal, to control external resorption processes, when the pulp shows vitality.

## RESUMO

Este artigo tem por objetivo principal expor os fundamentos ou razões pelas quais, em casos de dentes com reabsorção dentária externa, incluindo as de origem ortodôntica, não se deve fazer o canal para tratá-la. Isso se faz apenas nos dentes com contaminação ou necrose pulpar, para remover a inflamação periapical induzida pelos produtos microbianos. Frente a casos de reabsorção dentária externa, as condutas devem premiar sempre a seguinte sequência: primeiro, identificar a causa com precisão; depois, planejar a forma de abordagem terapêutica e, por fim, adotar as condutas de forma muito bem fundamentada. As situações em que o tratamento endodôntico está indicado na terapêutica das reabsorções dentárias são quando houver: a) necrose pulpar por contaminação microbiana, b) necrose pulpar asséptica, c) metamorfose cálcica da polpa inicial e d) diagnóstico de reabsorção interna. Não se consegue, por via pulpar, controlar o processo reabsortivo que está ocorrendo na parte externa; afinal, as causas estão atuando no ligamento periodontal. Não há qualquer evidência que justifique fazer o tratamento endodôntico, via canal, para controlar processos reabsortivos externos, quando a polpa está com vitalidade.

Contemporary Orthodontics interacts with all specialties and requires some basic knowledge of each of them, including diagnostic and technical abilities. Formerly, each specialty was isolated from the others, but nowadays, transdisciplinarity is determining to professional success before society and before a patient, the main stage to reaching maximum improvement. 

Tooth resorptions are present in all clinical specialties. Its diagnosis and treatment plan require transdisciplinary knowledge in decision making about how to treat them: if they demand treatment or just monitoring after the cause is identified. 

## ENDODONTICS AND TOOTH RESORPTIONS

The professional that the dental market determined as responsible, clinically and theoretically trained, to diagnose, control, opine and treat tooth resorption cases is the endodontist. However, not all endodontists received proper training for this subject during their formation as specialists in a detailed and biologically founded way.

When it is stated that the specialist at tooth resorption is the endodontist, we must be as careful as possible not to convey the wrong idea according to which all cases of resorption should be endodontically treated. Nevertheless, even the cases that do not require a root canal approach must be broadly mastered by the endodontist. 

Only part of the cases of tooth resorption should be exposed to treatment with root canal, especially the cases listed below:

a) pulp necrosis by microbial contamination;
b) aseptic pulp necrosis;
c) initial calcific metamorphosis of the pulp;
d) internal resorption.

## DENTAL PULP HAS NOTHING TO DO WITH EXTERNAL RESORPTIONS: THESE ARE THE FOUR WAYS OF STARTING A TOOTH RESORPTION

**Figure 1:**
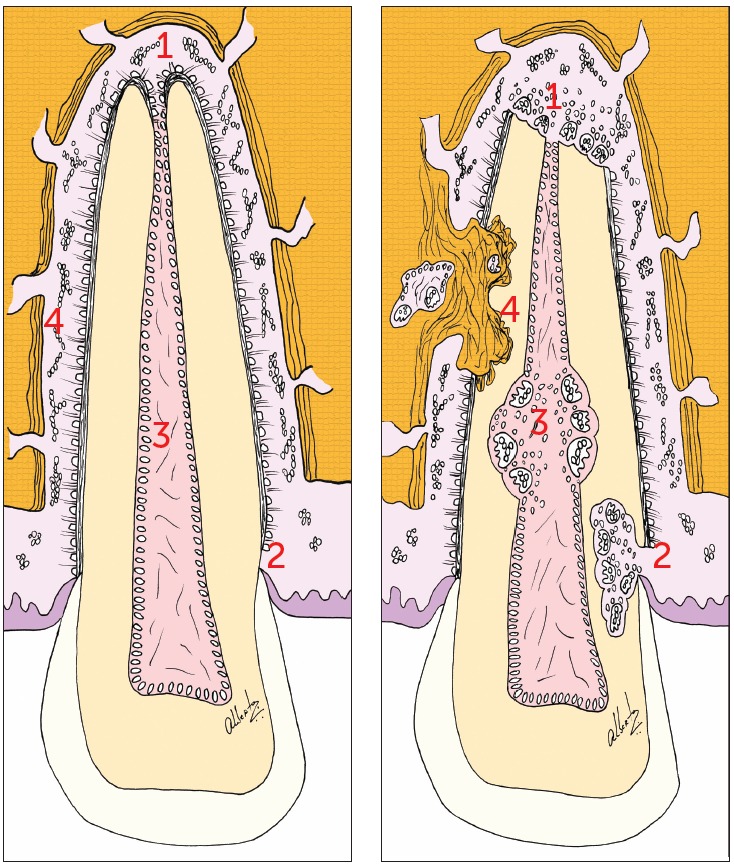
The destruction or exposure of the four structures that protect the teeth from resorption result in specific imaging scenarios which are characteristic of the types of tooth resorption. Automatically, this leads us to its cause, the treatment plan and prognosis.

Tooth resorptions may begin in four ways (Fig 1): 

1 - Cementoblasts and inflammatory external resorption.
2 - Gaps in the cementoenamel junction and external cervical resorption of inflammatory nature.
3 - Odontoblasts and internal resorption of inflammatory nature.

1) By destruction exclusively of the cementoblasts in the external part of the root, but leaving the other periodontal structures alive, although with varying inflammation degrees. 

In these cases, the resorption is classified as inflammatory external root resorption. The clinical causes that lead to this type of resorption are listed now in an order determined by their frequency: orthodontic movement, chronical periapical lesion, occlusal trauma and dental trauma of small magnitude. The most impaired region is the apical third.

When it comes to this type of tooth resorption (inflammatory external root resorption), if the cause is extinguished and this inflammation disappears, the process will be interrupted. Thus, the mediators will be greatly reduced and, in the process repair, the stem cells of the periodontal ligament and the pre-cementoblasts will restore the cementoblastic layer, so that the radicular mineralized structure will be again protected from the action of clasts. 

The professional's conduct and the treatment in these cases must privilege identifying the cause in order to control and eliminate it. Thus:

a) If the cause is not a chronical periapical lesion, which is almost always associated to pulp necrosis and root canal contamination, with bacteria and their products leaving through the apical foramen, there is no reason to provide endodontic treatment by means of root canal and its obturation.
b) If the cause is orthodontic movement, it is recommended to evaluate forces distribution, the movement intensity and its extension, besides other variables in the context of orthodontic treatment. Endodontic treatment is never recommended for inflammatory external root resorptions associated to orthodontic treatment.

Once one of these orthodontic variables is identified and controlled, the process naturally tends to be interrupted and the affected surface receives layers of cementoblasts and cementum covering the dentine, as it used to be. In case the process does not cease, one should reevaluate the identification of the cause in orthodontic context, but endodontic treatment should never be applied. Pulp will exhibit vitality and will not take part in the process, either directly or indirectly, and eliminating it would not be reasonable!

**Table 1:** Root resorptions by the death exclusively of cementoblasts, with maintenance of the epithelial remains of Malassez.

1.	Apical external inflammatory resorptions in the orthodontic movement.
2.	Apical external inflammatory resorptions in chronical periapical lesions.
3.	Apical external inflammatory resorptions in occlusal traumas.
4.	External inflammatory resorptions by small traumas

c) If the identified cause is occlusal dental trauma - the interferences, the forces eccentric load and/or traumatic occlusion -, it must be corrected, so the process will cease. Endodontic treatment by means of canal is not required when it comes to inflammatory external resorptions associated to lesion of occlusal trauma. That requires a diagnostic and therapeutical approach well based on knowledge about lesions of occlusal trauma.

We must highlight the fact that there is no methodologically acceptable evidence in the literature to reveal the possibility of the occlusal trauma inducing pulp necrosis. Whenever this diagnosis hypothesis exists, the professional must check for previous history of dental trauma in the case in question.

d) If the identified cause is due to dental traumas of lower magnitude, such as concussion, the resorption process will go backwards without symptoms. When diagnosed by imaging, it will have been in routine examinations. That is when it is possible to notice that the root surface is regular and uniformly and parallelly followed by periodontal uniform spaces and a hard layer equally regular and continuous. That characterizes a paralyzed tooth resorption.

In such situation, the professional must check for pulp vitality and thoroughly analyze the pulp space, in order to reject the possibility of processes that would be simultaneous and independent on external tooth resorptions, which are, respectively, aseptic pulp necrosis and pulp calcific metamorphosis (metaplasia). 

If none of these pulpopathies induced by dental trauma is found, then nothing justifies endodontic treatment for this type of paralyzed inflammatory external tooth resorption.

2) By exposure of dentine 'gaps' or windows in the cementoenamel junction, leaving alive the other gingival structures that cover it, although with inflammation degrees. All human teeth have these gaps in the external circumference of the cementoenamel junction, and its gaps are Achilles' heel of human teeth.

In these cases, the resorption will be classified as external cervical resorption and it has inflammatory nature. The clinical causes that induce this type of resorption are: dental trauma and internal tooth bleaching. Both may harm the gingival tissues that normally cover the dental cervix. 

The inflammation induced in the area ends up dissolving the gelled extracellular matrix of the gingival connective tissue and exposes the dentine to macrophage and other associated cells, which start the immunological recognizing process that culminates with its resorption.

The dental trauma that prevails as the cause of these cases is concussion, which the patient does not remember to report, due to its frequency and for it has no symptoms. When it comes to concussion, there is no increase of tooth mobility and, when there is a symptom, it is nothing but a brief uncomfortable sensation that lasts between 2 and 4h. All in all, it refers to small impacts or daily traumas. 

After the external cervical resorption begins, the process will hardly go backwards: it will be continuous, slow and will gradually compromise the cervical structure of the affected tooth. This type of tooth resorption has no etiopathogenic relation with the tooth pulp.

The professional's conduct and the treatment in these cases involve identifying the entry point of the resorption in the mineralized tissues and, once a gingival flap is raised, softly curetting the reabsorbed region, irrigating, protecting the pulp and filling with esthetically favorable material that must be compatible with the gingival tissues' health. The prognostic tends to be very good, unless there is evident physical fragilization of the tooth structure.

Some considerations on this type of external cervical resorption must be made:

a) The tooth tissues related to resorptions are not softened nor darkened. Differently from cavities, the affected tissues exhibit normal color and rigidity. When preparing the region to receive the material, vigorous curettage is not required, for there is no soften, darkened or contaminated tissue to be removed.
b) When closing a window or entry point of the process on the radicular surface, any nutrition or chance of the resorption process to continue is extinguished, for the vessels have been cut and there is no communication with the pulp.
c) The pulp, in these cases, is completely normal, without pulpitis or necrosis. The resorption process does not take acids and toxic products to the pulp through the dentinal tubules. The entire resorption process takes place in the clasts/dentine interface, without overflow or permeation of products and mediators to the surroundings or in depth.
d) In the treatment of external cervical resorption, there is no biological need to remove the pulp and provide endodontic treatment. That is justifiable only for technical reasons. The resorption may happen to be very profound and broad, besides being too close to the pulp, to the extent of being technically impossible to protect it or not drill the thin pulp wall which isolates and protects it. Even in the most profound and broad cases, the pulp will be normal, free of the any sign of pulpitis!
e) External tooth bleaching does not cause external cervical resorption. Internal tooth bleaching results in external cervical resorption in approximately 10% of the teeth exposed to it. In the cases of external cervical resorption induced by internal bleaching, when severe fragilization is not verified, the conduct should correspond to raising a gingival flap and filling with esthetically favorable material, which must be compatible with the gingival tissues.
f) Prognosis of external cervical resorption treatment in teeth with maintenance of pulp vitality or cases of internally bleached teeth is very positive. Recurrence cases have not been reported.

**Table 2:** Tooth resorptions by the direct exposure of the dentine to the gingival connective tissue in the gaps of the cementoenamel junction.

1.	Inflammatory external cervical resorption by accidental trauma, especially concussion.
2.	Inflammatory external cervical resorption by transurgical dental traumas (to expose non-erupted canines, for example, or during general anesthesia.
3.	Inflammatory external cervical resorption associated to internal bleaching.

3) By destruction exclusively of the odontoblasts in the external part of the root, but leaving the other pulpal structures alive, although with varying inflammation degrees.

In these cases, the resorption is classified as internal tooth resorption and it has inflammatory nature. The clinical cause of this type of resorption is dental trauma of small magnitude, especially concussion, which may be accidental or iatrogenic. 

Only in these less serious cases of trauma, such as concussion, the pulp will remain alive and the clasts may stay in the regions where the sudden movement of dental trauma displaced small areas of the odontoblastic layer, placing them at the center of the pulp. Without alive pulp there is no active internal resorption.

When it comes to internal resorption, endodontic treatment represents the only therapeutic conduct, and the prognosis is excellent, as long as there is no significant degree of structural fragilization. 

4) By simultaneous destruction of the epithelial remains of Malassez and cementoblasts in the external part of the root, with necrosis or elimination of the periodontal ligament in vast areas of the root surface, for the trauma leans the tooth against the surrounding bone tissue, scratching it. In these areas, the bone cells, bone marrow, vessels and endosteal tissues will be in close contact with the exposed mineralized radicular cementum.

When repairing these areas - in which the periodontal ligament is no longer alive or has been eliminated, for leaning against the alveolar bone, as a result of dental trauma -, the blood clot which fills it will be in a few hours colonized or invaded by vascular cells, leukocytes, fibroblasts, osteoblasts and clasts that come from immediately surrounding medullar-osseous spaces. The vessels and cells from the periodontal ligaments are farther, so it takes them longer to reach the area, comparing to the those from the bone tissue.

When the adjacent and distant epithelial remains of Malassez finally define they should migrate to that area, the periodontal space will have already been taken by components of osseous nature and origin, with precocious production of bone matrix at varying degrees of mineralization. In other words, the alveolodental ankyloses will be already established and, in some days, this newly-formed bone will start its continuous remodeling process, which includes the tooth that has been fused to the bone. Hence, the external tooth resorption by substitution will be established!

**Table 3:** Tooth resorptions by the death of odontoblasts, with pulp vitality maintenance.

1.	Internal inflammatory tooth resorption by accidental or iatrogenic dental trauma or iatrogenic, especially concussion.

**Table 4:** Root resorptions by the death of epithelial remains of Malassez and cementoblasts.

1.	Tooth resorptions by substitution induced by dental trauma.
2.	Root resorptions by substitution due to atrophy of the periodontal ligament in not erupted teeth, especially canines.

In external tooth resorption by substitution, there is no pulp impairment in the process. This type of tooth resorption has no etiopathogenic relation with the dental pulp. In case the pulp has pulp aseptic necrosis or calcific metamorphosis, it was due to the dental trauma, and not the external root resorption by substitution.

Endodontic treatment by means of canal cannot interfere in the process of osseous remodeling, which is being guided by inflammatory local factors and by systemic mediators, especially parathormone, calcitonin, vitamin D3 and estrogen. 

The prognosis of external tooth resorption by substitution is bad, and it will inevitably lead to tooth loss. An endodontic approach will not have positive influence over this prognosis. There is not even evidence of its influence over the time or pace of the tooth replacement by bone.

In teeth not erupted for long periods, atrophy of the periodontal ligament may lead to a risky approximation to the bundle bone and it may result in alveolodental ankylosis and, eventually, in resorption by substitution. This clinical situation is more common to the upper not erupted canines after years of intraosseous stage. 

## THE TWO MECHANISMS OF MINERALIZED TOOTH TISSUE RESORPTION

There are four ways of starting a tooth resorption, or four points at which the resorption process can be started (Fig 1). It is possible to affirm that a tooth has four vulnerable points for resorptions or four key-points that protect them from resorptions: cementoblasts, cementoenamel junction, odontoblasts and epithelial remains of Malassez. For their turn, the mechanisms that act in the resorption of the tooth tissue are two: 

1. The clasts act stimulated by mediators of the inflammatory process associated to an area that is deprived of mineralized tooth tissue and, which is, for that reason, called inflammatory mechanism of tooth resorption or inflammatory tooth resorption.

2. The clasts act upon the tooth tissues when they are attached to the bone and its mineralized parts which are, for their turn, steadily undergoing remodeling, including the teeth, in this constant formation/resorption/formation which characterizes our skeleton. This will only happen when there is alveolodental ankylosis: it is the mechanism of tooth resorption by substitution.

## FINAL CONSIDERATIONS

In external tooth resorptions, including those of orthodontic origin, the dental pulp does not take part in the process, in any situation. Only in the internal tooth resorption the pulp acts directly in the process.

One cannot control the resorption process that is taking place at the external part through the pulp, after all, the causes are acting upon the periodontal ligament. There is no evidence that justifies applying endodontic treatment, by means of canal, to control the external resorption processes when the pulp shows vitality.

Root canals are not performed to treat external tooth resorptions. That should be done only for contaminated teeth or those with pulp necrosis, in order to remove the periapical inflammation induced by microbial products. When facing cases of external tooth resorption, the conducts must always prioritize the following sequence of steps: firstly, identifying the cause accurately; after that, planning the therapeutic approach and, finally, adopting conducts in a very well-founded way.
